# Barcode sequencing: a robust, platform-agnostic method for massively parallel cell-based screens

**DOI:** 10.1093/g3journal/jkaf166

**Published:** 2025-07-18

**Authors:** Marjan Barazandeh, Hamid Kian Gaikani, Rutuja Pattanshetti, Joseph Uche Ogbede, Sunita Sinha, Rachel Moore, Christopher E Carr, Guri Giaever, Corey Nislow

**Affiliations:** Pharmaceutical Sciences, The University of British Columbia, 2405 Wesbrook Mall, Vancouver, BC, Canada V6T 1Z3; Pharmaceutical Sciences, The University of British Columbia, 2405 Wesbrook Mall, Vancouver, BC, Canada V6T 1Z3; Pharmaceutical Sciences, The University of British Columbia, 2405 Wesbrook Mall, Vancouver, BC, Canada V6T 1Z3; Pharmaceutical Sciences, The University of British Columbia, 2405 Wesbrook Mall, Vancouver, BC, Canada V6T 1Z3; UBC Sequencing and Bioinformatics Consortium, Pharmaceutical Sciences, The University of British Columbia, 2405 Wesbrook Mall, Vancouver, BC, Canada V6T 1Z3; Daniel Guggenheim School of Aerospace Engineering, School of Earth and Atmospheric Sciences, Georgia Institute of Technology, North Avenue, Atlanta, GA 30332, United States; Daniel Guggenheim School of Aerospace Engineering, School of Earth and Atmospheric Sciences, Georgia Institute of Technology, North Avenue, Atlanta, GA 30332, United States; Pharmaceutical Sciences, The University of British Columbia, 2405 Wesbrook Mall, Vancouver, BC, Canada V6T 1Z3; Pharmaceutical Sciences, The University of British Columbia, 2405 Wesbrook Mall, Vancouver, BC, Canada V6T 1Z3

**Keywords:** Bar-seq, functional genomics, high-throughput sequencing, yeast genomics, HIPHOP, *Saccharomyces cerevisiae*, drug assay, fungi

## Abstract

Barcode sequencing (Bar-seq) is a high-throughput method originally developed for systematically identifying gene–drug interactions and genetic dependencies in yeast using pooled deletion-mutant libraries. This approach enables high-resolution profiling of large mutant libraries over time, across diverse experimental conditions, providing relative fitness values for each individual within the population. As the technology for enumerating barcodes has evolved, we have continued to incorporate improvements to the method. Here, we present an optimized Bar-seq workflow adaptable to multiple sequencing platforms, including instruments from Illumina, MGI, Element, and Oxford Nanopore. We highlight the advantages and limitations of each approach to aid in experimental design decisions. We introduce refinements in barcode amplification, sequencing strategies, and data analysis to enhance accuracy and scalability while making adoption as straightforward as possible.

## Teaser

This study provides a comprehensive and accessible guide to Barcode Sequencing (Bar-seq), a powerful method for investigating gene-by-environment interactions across thousands of strains in parallel. Aimed at researchers in functional genomics, especially graduate trainees, Barazandeh *et al*. present detailed, platform-agnostic protocols for Bar-seq library preparation, sequencing, and data analysis. The authors also benchmark data quality across multiple sequencing platforms, offering practical insights into platform selection. Their results highlight the value of Bar-seq in uncovering functional relationships and drug-gene interactions at scale. This work serves as a resource for expanding the utility of pooled genomic assays in diverse experimental settings.

## Introduction

*Saccharomyces cerevisiae* has been widely used as a eukaryotic model organism to study cellular processes relevant to multicellular organisms, including humans. These studies and the vibrant community of yeast researchers have advanced our understanding of the genome and of gene–gene and gene–environment interactions (Botstein et al. 1997; Giaever and Nislow 2014; Mohammad et al. 2018). In addition to being a model eukaryotic organism, many aspects of yeast research can be translated to metazoans. For example, human gene function may be inferred by virtue of homology (Dey et al. 2015), and in many cases, the human ortholog can be studied directly in yeast (Kachroo et al. 2022; Ólafsson et al. 2023). Compared to mammalian systems, yeast offers a rapid, cost-effective, and genetically manipulable platform for functional genomic studies (Botstein et al. 1997; Botstein and Fink 2011; Gaikani et al. 2024).

One powerful application of yeast genomics was realized by the development of the yeast knockout (YKO) collection. Completed in 2002, the YKO consists of over 21,000 deletion strains (Giaever et al. 2002). Each strain carries a stop-to-start deletion of an open reading frame (ORF), with essential genes deleted as heterozygotes and with nonessential genes deleted as homozygous diploids after sporulation and mating of haploids of both mating types (Mat a and Mat α). These deletions are marked by 2 unique 20-bp DNA barcodes (uptags and downtags), flanked by common primer sites such that all barcodes can be amplified *en masse* (Supplementary Fig. 1). Many of the experimental design and data analysis elements developed for the YKO have been incorporated into systematic mammalian cell screens (Hart et al. 2015; Wang et al. 2015).

One fruitful application of the YKO collection has been the development of HIPHOP (*H*aplo*I*nsufficiency *P*rofiling and *HO*mozygous *P*rofiling), a chemogenomic strategy that assesses drug–gene interactions by exposing both essential (heterozygous) and nonessential (homozygous) deletion pools to various small molecules and stress conditions (Hoepfner et al. 2014; Lee et al. 2014). The assay identifies potential drug targets by measuring fitness changes in response to treatment. For example, if a heterozygous deletion strain is hypersensitive to a compound, the targeted gene is likely essential for growth in the presence of that drug (HIP assay). Conversely, if a homozygous deletion strain shows sensitivity, the deleted gene may play a role in buffering the drug's effect (HOP assay).

Initially, the HIPHOP assay employed barcode microarrays, where barcodes amplified from pools of the YKO were hybridized to microarrays containing oligonucleotides corresponding to the barcode complements either spotted onto a glass surface or synthesized via photolithography (Shoemaker et al. 1996 ; Lum et al. 2004; Pan et al. 2004; Pierce et al. 2007; Ammar et al. 2009). Over time, microarray-based assays and analysis improved, with increases in feature density and decreases in feature size, including Agilent microarrays (Pan et al. 2004), Affymetrix TAG1 (Shoemaker et al. 1996), TAG3 (Giaever et al. 2002), TAG4 (Pierce et al. 2007), and *S. cerevisiae* whole genome tiling arrays (Ammar et al. 2009). The advent of next-generation sequencing (NGS) disrupted the field, and the introduction of barcode sequencing (Bar-seq) proved to be a cost-effective and scalable alternative to barcode microarrays (Smith et al. 2009, 2010). In the decade since its introduction, the amount of data that may be obtained from a single Bar-seq flow cell has increased over 3 orders of magnitude—from 1 million NGS reads to >10 billion.

In addition to dramatic increases in throughput compared to microarrays, Bar-seq provides improved sensitivity, dynamic range, and reproducibility. Notably, Bar-seq was used to correct YKO barcode misannotation, revealing that nearly 20% of barcodes deviated from their original assignments, leading to higher-quality data for genetic analysis (Smith et al. 2009). Finally, since Bar-seq does not require that the content be defined a priori, any pool of barcodes, including those with random sequences, is a suitable substrate for experimentation.

With the rapid evolution of NGS, Bar-seq has been iteratively optimized to accommodate newer platforms and to improve barcode counting accuracy (Smith et al. 2010; Robinson et al. 2013 ; Acton et al. 2017). While early implementations relied primarily on Illumina GAII and HiSeq instruments, we have since adapted Bar-seq to other platforms, such as Illumina NextSeq and NovaSeq, MGISEQ, Element, and Oxford Nanopore instruments. The ability to use diverse platforms provides flexibility. The work here compares the performance of these platforms, discusses different use cases, and highlights best practices for each platform to ensure cross-platform reproducibility of the data.

Beyond drug assays, Bar-seq has also proven to be a powerful tool for a wide range of functional genomic studies in yeast, including environmental perturbation experiments, epigenomic studies, and long-term evolution experiments (Hillenmeyer et al. 2008; Kollenstart et al. 2021; Li et al. 2023). Its versatility makes it a valuable method for exploring diverse genetic and phenotypic interactions at scale, such as detecting protein–protein interactions in vivo in pooled yeast strains using Barcode Fusion Genetics-Yeast 2-Hybrid (BFG-Y2H) and mapping dynamic interaction networks through a high-throughput, double-barcoding system (Yachie et al. 2016; Schlecht et al. 2017). Additionally, Bar-seq has been applied to identify genes essential for fitness in other yeast species and organisms, including the fungal pathogen *Candida albicans*, where a pooled screening approach enables the quantification of strain-specific barcodes to assess fitness under diverse environmental conditions (Xiong et al. 2025). Similarly, in the bacterium *Vibrio fischeri*, a Bar-seq-based method integrates targeted gene deletion with short-read sequencing to study microbial colonization dynamics and mixed population behavior (Burgos and Mandel 2024). Below, we provide an optimized workflow for Bar-seq across different sequencing platforms, incorporating refinements in barcode amplification, library preparation, and data analysis.

## Materials and methods

### Media preparation and yeast growth

Yeast cells were cultured in synthetic complete (SC) medium prepared by dissolving 0.17 g of BD Difco yeast nitrogen base without amino acids and ammonium sulfate (Fisher Scientific, cat# DF0335-08-8), 0.5 g of ammonium sulfate (Fisher Scientific, cat# A702-500), 0.2 g of SC amino acid supplement powder (Sunrise Science Products, cat# 1300-030), and 2.0 g of dextrose in distilled water, with the final volume adjusted to 100 mL. The medium was sterilized by filtration and stored at room temperature.

The starting OD600 of cultures was set to 0.0625, and cells were grown to late log phase (∼1.2 OD600), corresponding to ∼5 generations of growth before the cultures reach saturation.

All assays were conducted using fully automated robotic liquid handling systems (S&P Robotics Inc., Toronto, ON, Canada, and a BioTek LogPhase 600 plate reader from Agilent). Yeast cultures were grown in 48-well or 96-well flat-bottom plates (Thermo Scientific, cat# 130187 and Corning, cat# 3598, respectively) at 30 °C with intermittent shaking. Growth was monitored by measuring OD600 every 10 to 15 min. Drug/small molecule treatments were applied at a final concentration of 2% DMSO, with appropriate DMSO or water controls run in parallel (Pierce et al. 2007).

### Bar-seq workflow

The Bar-seq workflow consists of several components: a bioassay, HIPHOP assay, and DNA extraction, barcode amplification and sequencing, and data analysis, including fitness defect (FD) score calculation and functional enrichment analysis.

#### Bioassay

To determine the dose–response of yeast cells to different drugs, the wild-type diploid strain BY4743 was exposed to at least 6 serial halving dilutions of each drug, spanning a range—from the highest concentration (depending on the solubility) to the lowest. Each condition was tested in triplicate. Control wells contained DMSO (up to 2% final concentration) or water, depending on drug solvent. In contrast to mammalian cells, yeast typically requires higher doses of compound to achieve growth inhibition, owing to their cell wall and robust efflux transporters, so we typically select a high starting dose (e.g. 2 mM or as high as possible given the compound solubility) for the serial dilutions. The use of relatively high drug doses is discussed in detail in Roemer et al. (2012).

Drug potency was determined by calculating IC20 values (the concentration at which cell growth is inhibited by 20% relative to the control) after 5 generations (∼16 h at 30 °C). Growth kinetics, inhibition percentages, and doubling times were analyzed using the AUDIT platform (https://ggshiny.shinyapps.io/AUDIT-geneticnetworks/) (Coutin et al. 2020).

#### HOP assay

The homozygous deletion pool (∼4,800 strains) from the YKO collection was grown in the presence of drugs or controls at their IC20 concentrations for 5 generations. Cells were then harvested and stored at 4 °C prior to genomic DNA extraction.

#### HIP assay

The essential heterozygous deletion pool (∼1,200 strains) was similarly grown under drug or control conditions as above, but for 20 generations. This extended treatment allows for the detection of modest strain sensitivity—the limit of detection is such that an ∼3% FD relative to controls can be reliably measured. Since cells reach saturation after 5 generations, they were diluted into fresh media containing the same IC20 drug concentration every 5 generations (total of 3 transfers). The S and P robotic liquid handler facilitates these serial exposures, but they can be accomplished manually by diluting each culture once it reaches saturation (e.g. see Xu et al. 2007). After 20 generations, cells were collected robotically for DNA extraction.

#### Genomic DNA extraction

Genomic DNA was extracted using the YeaStar Genomic DNA Kit (Zymo Research, D2002) following the manufacturer's protocol. While we have found this product to be robust and reproducible for the past 2 decades, any high-quality gDNA protocol that efficiently extracts yeast gDNA should suffice. DNA quantity and purity were assessed using the Agilent Biotek Cytation 5 Take3 microplate reader.

#### Bar-seq library preparation

Bar-seq libraries were typically prepared through 2 rounds of PCR amplification. In PCR1, the extracted genomic DNA was used as a template to amplify uptag and downtag barcodes from the YKO pool using common flanking primers in separate reactions (1 for each tag). After PCR1, the amplified uptags and downtags were pooled equally and then used as the template for PCR2, where Illumina-compatible adapters were added to the amplicons.

We initially used the same Illumina-compatible amplicons (∼200 bp) for sequencing on the Oxford Nanopore MinION device. Subsequently, to further simplify sample processing, we took advantage of the Oxford Nanopore's ability to sequence long DNA fragments to implement a single-step PCR as described in the Nanopore section below.

#### Bar-seq by Illumina

**Fig. 1.**
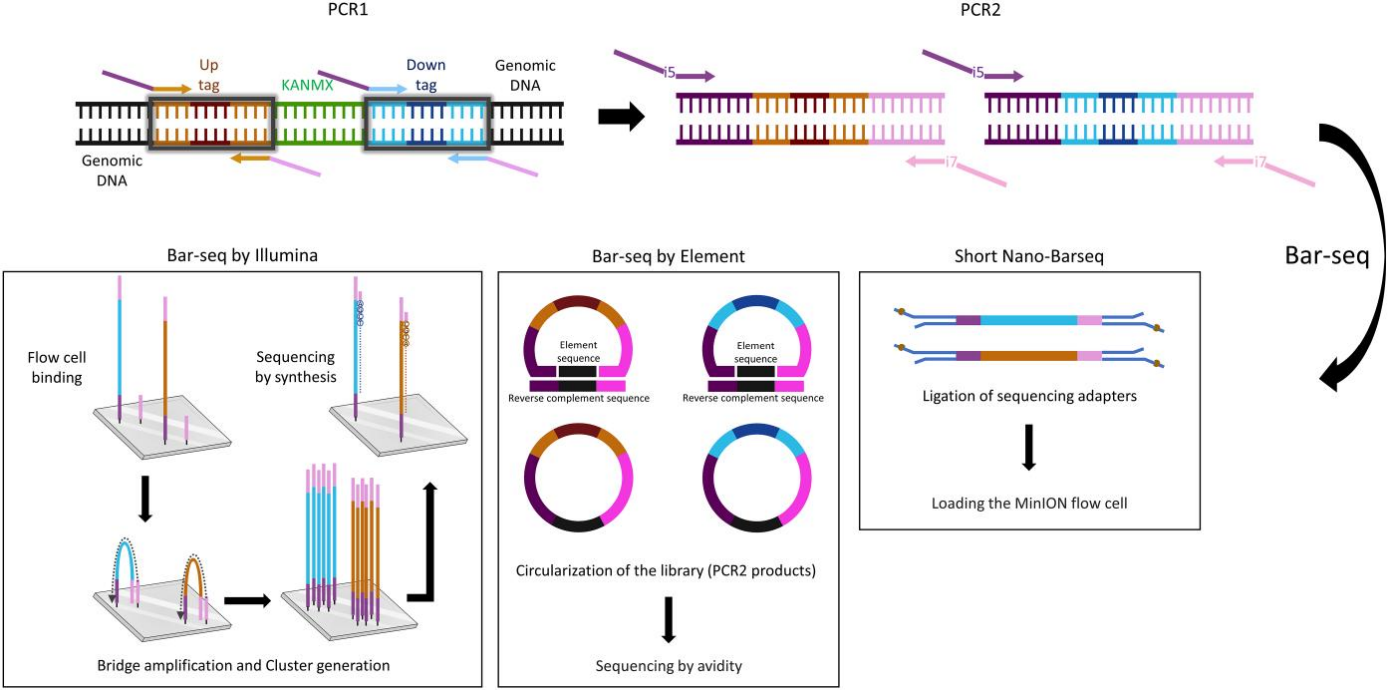
Bar-seq workflow using Illumina, Element, and Oxford Nanopore sequencing platforms. In PCR1, the deletion cassette regions containing the uptag and downtag barcodes, flanked by common priming sites, are amplified. The amplified products undergo PCR2, in which Illumina adapter and index sequences are added, to generate sequencing-ready libraries. In Bar-seq by Illumina, the library is introduced onto the flow cell as single molecules that bind by virtue of the complementary sequences added in PCR2. After binding, library molecules are subjected to bridge amplification and cluster generation and subsequently sequenced using the sequencing by synthesis approach. In Bar-seq by Element, the Illumina libraries are converted using the Adept Library Compatibility workflow, which uses the Illumina PCR2 products as input. These are then circularized, enabling sequencing by avidity. The short Nano-Barseq libraries are prepared using PCR2 products and the Ligation Sequencing Kit V14 (SQK-LSK114). The prepared library is then sequenced using a MinION device with MinKNOW software. Partially created in BioRender. Barazandeh, M. (2025), https://BioRender.com/aw66zb4.

The Bar-seq by Illumina consists of 2 rounds of PCR amplification (Fig. 1). PCR1 primers were designed to amplify uptag and downtag barcodes in the *S. cerevisiae* YKO collection, along with their common flanking regions. The Nextera Transposase Adapters (indicated in italic) were incorporated into the common primers (in bold):

- *S. cerevisiae* Illumina PCR1 uptag
  - 5′ *TCGTCGGCAGCGTCAGATGTGTATAAGAGACAG***GATGTCCACGAGGTCTCT** 3′
  - 5′ *GTCTCGTGGGCTCGGAGATGTGTATAAGAGACAG***GTCGACCTGCAGCGTACG** 3′
- *S. cerevisiae* Illumina PCR1 downtag
  - 5′ *TCGTCGGCAGCGTCAGATGTGTATAAGAGACAG***GAAAACGAGCTCGAATTCATCG** 3′
  - 5′ *GTCTCGTGGGCTCGGAGATGTGTATAAGAGACAG***CGGTGTCGGTCTCGTAG** 3′

To extend this approach to the fission yeast *Schizosaccharomyces pombe* deletion-mutant library, primers were designed based on published common sequences (Romila et al. 2021), with Nextera Transposase Adapters added to maintain compatibility with the *S. cerevisiae* YKO collection design:

- *S. pombe*_ Illumina PCR1 uptag
  - 5′ *TCGTCGGCAGCGTCAGATGTGTATAAGAGACAG*CGCTCCCGCCTTACTTCGCATTTAAA 3′
  - 5′ *GTCTCGTGGGCTCGGAGATGTGTATAAGAGACAG***GGGGACGAGGCAAGCTAAGATATC** 3′
- *S. pombe*_ Illumina PCR1 downtag
  - 5′ *TCGTCGGCAGCGTCAGATGTGTATAAGAGACAG***CGCCATCCAGTGTCGAAAAGTATC** 3′
  - 5′ *GTCTCGTGGGCTCGGAGATGTGTATAAGAGACAG***TTGCGTTGCGTAGGGGGGATTTAAA** 3′

PCR1 reactions were carried out in a final volume of 20 µL, containing 0.2 µL of Thermo Scientific Phusion Hot Start II DNA polymerase (2 U/µL), 4 µL of 5× Phusion HF buffer, 0.4 µL of 10 mM dNTPs, 50 to 100 ng of genomic DNA, and 0.5 µM of forward and reverse primers. The thermocycling conditions were as follows: initial denaturation: 3 min at 98 °C, 25 cycles of denaturation at 98 °C for 10 s, annealing at 59 °C for 30 s and extension at 72 °C for 20 s, and a final extension at 72 °C for 5 min. It is important to note that while we rely on Phusion Hot Start II DNA polymerase in our experiments, any high-fidelity polymerase should yield satisfactory results. In practice, we find that preparing large batches of each master mix and a large number of aliquots is important to ensure sample-to-sample reproducibility.

PCR1 products were examined via 1% agarose gel electrophoresis. Equimolar amounts of uptag and downtag amplicons were pooled, diluted 10-fold, and used in PCR2 to incorporate Illumina Nextera dual indexes (i7 and i5 adapters). The indexes are available as “IDT for Illumina UD Indexes” and can be ordered in bulk either from Illumina or from third-party oligo providers. The 3′ ends of the PCR2 primers (indicated in italics below) match the 5′ sections of the PCR1 primers for both *S. cerevisiae* and *S. pombe* mutant collections:

- Illumina PCR2 primers:
  - 5′ CAAGCAGAAGACGGCATACGAGAT**[i7]***GTCTCGTGGGCTCGG* 3′
  - 5′ AATGATACGGCGACCACCGAGATCTACAC**[i5]***TCGTCGGCAGCGTC* 3′

PCR2 was performed in 50 µL, containing 0.5 µL of Phusion Hot Start II DNA polymerase, 10 µL of 5× Phusion HF buffer, 1 µL of 10 mM dNTPs, 1.5 µL of 1/10 diluted PCR1 product, and 0.5 µM of PCR2 primers.

For the PCR2 conditions, the thermocycling conditions were as follows: 3 min at 98 °C, 6 cycles of denaturation at 98 °C for 30 s, and annealing at 60 °C for 30 s and extension at 72 °C for 30 s, followed by a final extension at 72 °C for 5 min.

PCR2 products, yielding a final fragment size of 200 bp, were purified using HighPrep PCR Clean-up System (1.8× beads:DNA), quantified via Qubit dsDNA HS kit, and analyzed using Agilent High Sensitivity D1000 ScreenTape. Libraries were normalized, pooled, and sequenced using Illumina NextSeq 550 (Single Read 50) or NovaSeq 6000-S4 (Paired End 150), targeting 5 to 6 million reads per sample. This number of reads will, in principle, provide ∼500 unique sequence reads/strain.

It is important to note that this 2-step PCR strategy is amenable to a wide variety of amplicon libraries. In principle, any collection of amplicons that have (or can be engineered with) common flanking sequences should be amenable to this approach.

#### Bar-seq by MGISEQ

**Fig. 2.**
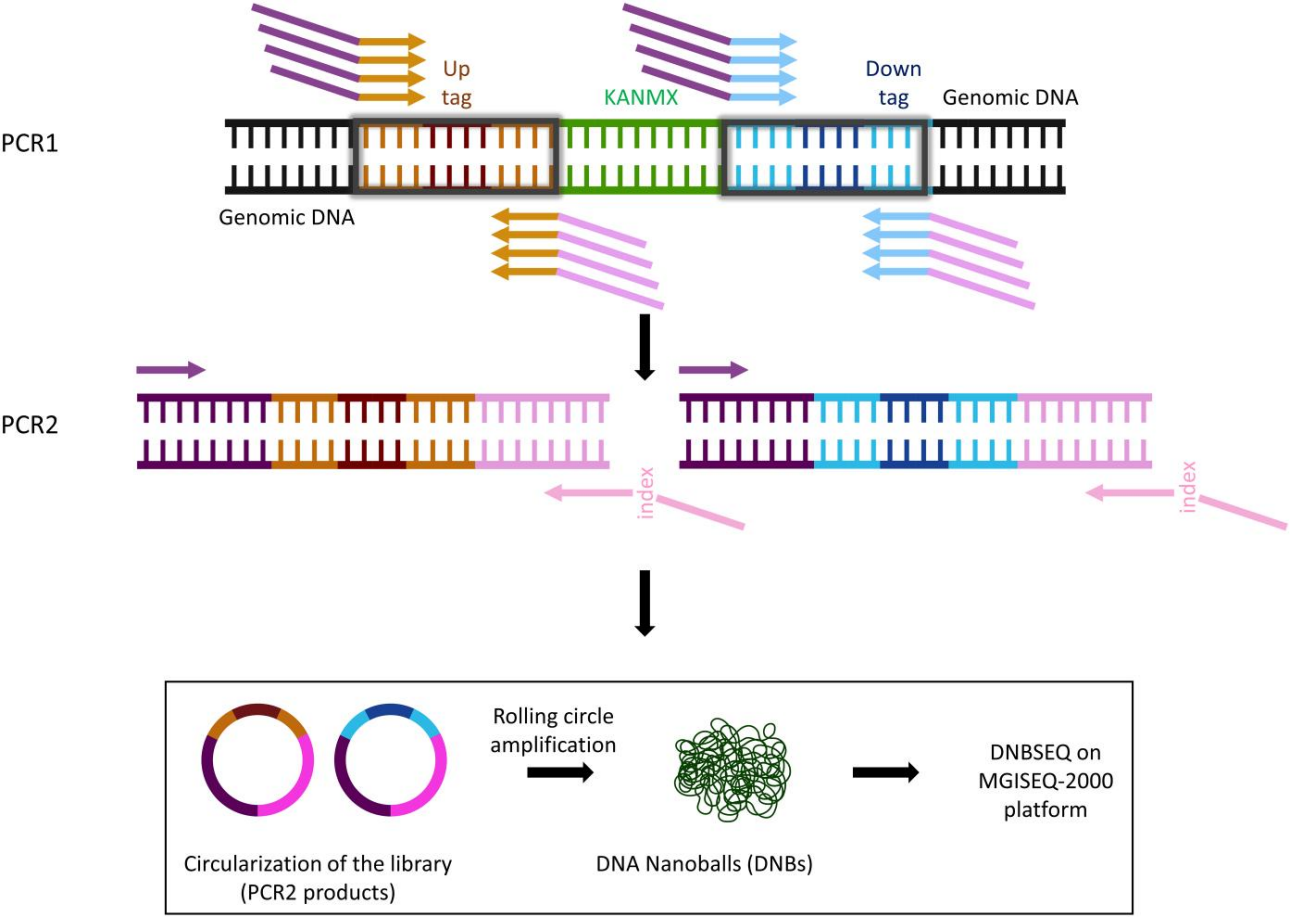
Bar-seq workflow adapted for the MGI sequencing platform. In PCR1, the deletion cassette containing the uptag and downtag barcodes, flanked by common priming sites, is amplified using a modification of the Illumina protocol. Specifically, the PCR1 primers are staggered, i.e. offset by 1 base per primer. This staggering minimizes the effect of low sequence diversity from common sequences and introduces base complexity for the initial reads, which is required for the MGI base-calling algorithms to accurately interpret sequencing signals. PCR1 products undergo a traditional PCR2, generating sequencing-ready libraries. The libraries are then circularized and amplified via rolling circle amplification to generate DNA nanoballs, which are subsequently bound to patterned flow cells and sequenced using DNBSEQ technology on the MGISEQ-2000 platform. Partially created in BioRender. Barazandeh, M. (2025), https://BioRender.com/xk7g24s.

Bar-seq on the MGISEQ platform is also performed using 2 separate PCR reactions, PCR1 and PCR2 (Fig. 2). PCR1 utilizes a total of 8 primers per reaction, consisting of 4 forward and 4 reverse primers. These primers are nearly identical, differing only by a single-base shift (shown in regular font below) after the MGIEasy sequencing primers (italicized) and just before the barcode-flanking common region (bolded). These staggered primers introduce base complexity for the common sequences, which is required for color balancing during imaging and for the MGI base-calling algorithms. The sequences for the PCR1 primers are listed below:

- MGISEQ PCR1 uptag
  Forward primers:
  - 5′ *GAACGACATGGCTACGATCCGACTT***GATGTCCACGAGGTCTCT** 3′
  - 5′ *GAACGACATGGCTACGATCCGACTT*C**GATGTCCACGAGGTCTCT** 3′
  - 5′ *GAACGACATGGCTACGATCCGACTT*GA**GATGTCCACGAGGTCTCT** 3′
  - 5′ *GAACGACATGGCTACGATCCGACTT*TGC**GATGTCCACGAGGTCTCT** 3′
  Reverse primers:
  - 5′ *CCGCTTGGCCTCCGACTT***GTCGACCTGCAGCGTACG** 3′
  - 5′ *CCGCTTGGCCTCCGACTT*A**GTCGACCTGCAGCGTACG** 3′
  - 5′ *CCGCTTGGCCTCCGACTT*TC**GTCGACCTGCAGCGTACG** 3′
  - 5′ *CCGCTTGGCCTCCGACTT*CAC**GTCGACCTGCAGCGTACG** 3′
- MGISEQ PCR1 downtag
  Forward primers:
  - 5′ GAACGACATGGCTACGATCCGACTT**GAAAACGAGCTCGAATTCATCG** 3′
  - 5′ *GAACGACATGGCTACGATCCGACTT*A**GAAAACGAGCTCGAATTCATCG** 3′
  - 5′ *GAACGACATGGCTACGATCCGACTT*TC**GAAAACGAGCTCGAATTCATCG** 3′
  - 5′ *GAACGACATGGCTACGATCCGACTT*CAC**GAAAACGAGCTCGAATTCATCG** 3′
  Reverse primers:
  - 5′ *CCGCTTGGCCTCCGACTT***CGGTGTCGGTCTCGTAG** 3′
  - 5′ *CCGCTTGGCCTCCGACTT*C**CGGTGTCGGTCTCGTAG** 3′
  - 5′ *CCGCTTGGCCTCCGACTT*GA**CGGTGTCGGTCTCGTAG** 3′
  - 5′ *CCGCTTGGCCTCCGACTT*TGC**CGGTGTCGGTCTCGTAG** 3′

An equimolar mix of 4 primers was prepared before PCR1. The PCR1 reactions followed the same conditions as the Illumina Bar-seq protocol. After amplification, uptag and downtag reactions were pooled in equal proportions, and a 1/10 dilution was used as the template for PCR2, which incorporated MGI adapters and indexes into the amplified PCR1 fragments. MGISEQ employs a single indexing method, requiring only 1 PCR2 forward primer that aligns with the 5′ ends of the PCR1 forward primers. The reverse primers include sample-specific unique indexes from the MGIEasy index system, with their 3′ ends (italicized below) complementing the 5′ ends of the PCR1 reverse primers:

- 5′ *GAACGACATGGCTACGA* 3′
- 5′ TGTGAGCCAAGGAGTTG**[index]**TTGTCTTCCTAAGA*CCGCTTGGCCTCCGACTT* 3′

MGI PCR2 reactions were performed in a final volume of 25 µL, containing 0.25 µL of Thermo Scientific Phusion Hot Start II DNA polymerase (2 U/µL), 5 µL of 5× Phusion HF buffer, 0.5 µL of 10 mM dNTPs, 50 to 100 ng of genomic DNA, and 0.5 µM forward and reverse primers. Thermocycling conditions were as follows: 8 cycles of denaturation at 98 °C for 30 s and annealing at 60 °C for 30 s and extension at 72 °C for 30 s, followed by a final extension at 72 °C for 5 min. The amplified PCR2 products were purified using AMPure XP beads (1.8× beads:DNA), and fragment size (∼200 bp) was confirmed using the Agilent 2100 Bioanalyzer. DNA concentration was measured with the Qubit dsDNA High Sensitivity (HS) assay. A total of 0.5 pmol of each library was pooled based on unique sample indexes. The pooled libraries were then converted to single-stranded circular DNA using the MGIEasy Circularization Kit and sequenced on the MGISEQ-2000 platform (software version: 1.0.8.208), incorporating a 5% control spike-in DNA. Sequencing was performed in a single-end (SE) 50-bp format. The first 10 cycles were dark cycles, followed by 40 cycles of sequencing, yielding a total of 50 bases per SE read.

#### Bar-seq by element

Library preparation for Element AVITI sequencing followed the same steps as Bar-seq by Illumina, using the Element Adept Library Compatibility Workflow (Fig. 1). This workflow enables the circularization of libraries prepared with third-party assays while preserving barcode integrity. The process involves annealing a long and short splint oligo to facilitate adapter integration, ensuring compatibility with the AVITI system while retaining original indexes and adapters.

Sequencing was performed on the AVITI platform from Element Biosciences (both SE and paired-end 150 bp reads) using the following custom sequencing primers to avoid conserved regions within the sequences (common uptag and downtag primers):

uptag_R1: 5′ GTATAAGAGACAGGATGTCCACGAGGTCTCT 3′

uptag_R2: 5′ TATAAGAGACAGGTCGACCTGCAGCGTACG 3′

downtag_R1: 5′ AGAGACAGGAAAACGAGCTCGAATTCATCG 3′

downtag_R2: 5′ GTATAAGAGACAGCGGTGTCGGTCTCGTAG 3′

#### Nano-Barseq by Oxford Nanopore

Nanopore-based Bar-seq (Nano-Barseq) can be performed in 2 formats: short Nano-Barseq and long Nano-Barseq.

Short Nano-Barseq uses the same 2-round PCR protocol and primers as the Illumina Bar-seq workflow (Fig. 1).

**Fig. 3.**
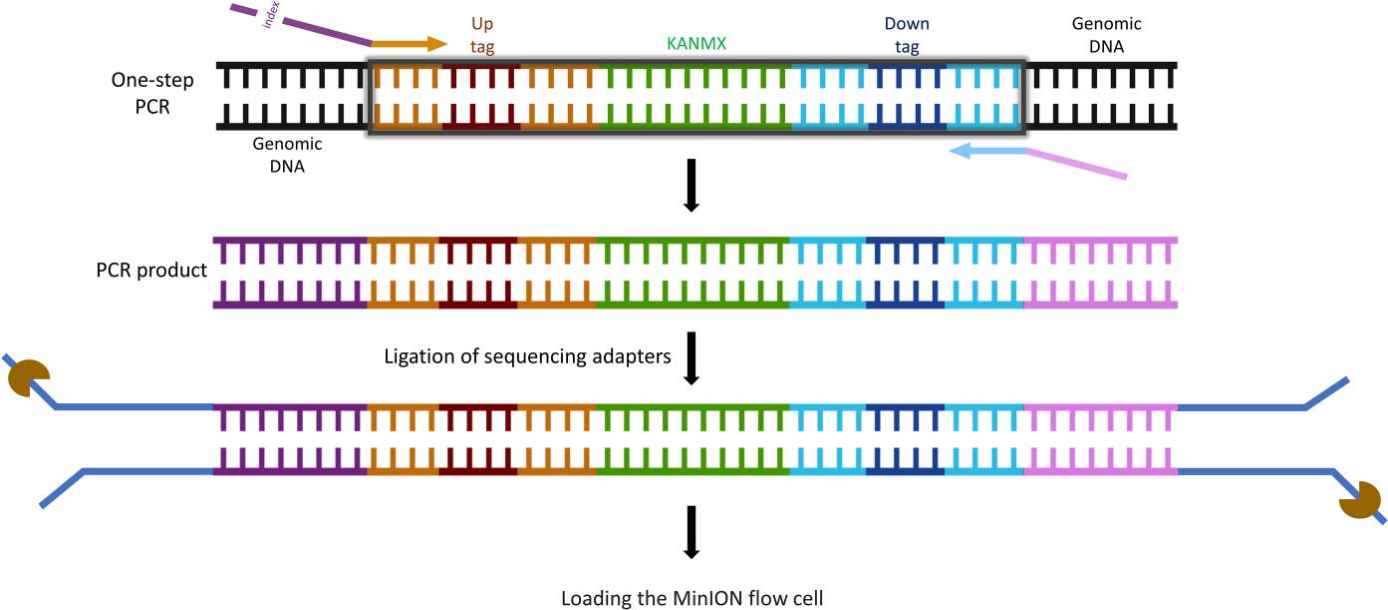
Nano-Barseq workflow using Oxford Nanopore’s MinION platform for sequencing. A 1-step PCR is performed using primers that amplify the entire deletion cassette, including both uptag and downtag barcodes, their flanking common primers, and the KANMX selection marker. The resulting ∼1,600-bp PCR product is directly used for library preparation, utilizing the Ligation Sequencing Kit V14 (SQK-LSK114), which enables high-fidelity sequencing of long DNA fragments. The prepared library is then sequenced using MinION with MinKNOW software. Partially created in BioRender. Barazandeh, M. (2025), https://BioRender.com/lu5ld6s.

In contrast, long Nano-Barseq is a streamlined, single-step PCR method optimized for Oxford Nanopore sequencing, eliminating the need for a second amplification round (Fig. 3). This approach enables direct sequencing of both barcode regions in a single read, reducing sample preparation time and costs. A single round of PCR was performed using the PCR1 uptag forward primer and the PCR1 downtag reverse primer, amplifying the entire genomic locus (∼1,600 bp) that includes both the uptag and downtag.

In this setup, only the uptag receives a sequencing index. To reduce sequencing noise, a 5′ common 15-mer sequence (GCTAGTACGTGACAT) was added directly upstream (5′) of the unique indexes. Predesigned indexes (Xu et al. 2009), shortened from 25-mers to 10-mers to align with standard Illumina indexing conventions, were appended immediately downstream of this common sequence. The Illumina PCR1 uptag primer was then added to the 3′ end of this composite sequence, enabling amplification of the barcoded region. The downtag primer remained unchanged from the standard Illumina PCR1 downtag primer.

PCR was performed in a final volume of 50 µL, containing 0.5 µL of Thermo Scientific Phusion Hot Start II DNA polymerase (2 U/µL), 10 µL of 5× Phusion HF buffer, 1 µL of 10 mM dNTPs, 50 to 100 ng of genomic DNA, and 0.5 µM of forward and reverse primers. The thermocycling conditions were as follows: initial denaturation: 3 min at 98 °C, 25 cycles of denaturation at 98 °C for 10 s, annealing at 59 °C for 30 s and extension at 72 °C for 90 s, and a final extension at 72 °C for 10 min.

The rest of the library preparation process was performed similar to the Illumina platform, and samples were sequenced on a MinION flow cell (serial # FBA16143). PCR products were stabilized with DNA/RNA Shield (Zymo Research R1200) for shipping and purified using column cleanup (Zymo Research D4014 DNA Clean & Concentrator-5) with a modification of heating the elution buffer to 80 °C prior to elution. Libraries were prepared using the Ligation Sequencing Kit V14 (SQK-LSK114revE_29Jun2022) in short fragment mode with the recommended BSA additive, targeting 250 fmol input, with a yield of 68%. Adapter ligation followed, and sequencing was performed over 72 h with basecalling turned off. Basecalling and adapter trimming were conducted using ONT Dorado v0.7.3, followed by quality control analysis with pycoQC. The run yielded 9.24 million reads and 14.8 Gbp of data (97% pass reads), with a median Phred score of 21.0 (21.2 for pass reads).

### Statistical analysis

Bar-seq reads were aligned to the YKO barcode reference database using BWA v0.7.17 (Li and Durbin 2009). Reads were filtered using SAMtools v1.9 (Danecek et al. 2021), retaining only those with a mapping quality ≥ 30. Read counts were quantified using BEDTools v2.29.1 (Quinlan and Hall 2010), and differential fitness scores were calculated using various R packages (dplyr, tidyr) (Wickham et al. 2018; Barazandeh et al. 2022; Wickham and Girlich 2022). Gene set enrichment analysis and network visualizations were performed using GSEA (v.4.2.3) and Cytoscape (v.3.10.3) (Subramanian et al. 2005; Gustavsen et al. 2019).

An important consideration in analyzing Bar-seq data is whether or not the uptag and downtag data should be analyzed separately or together. For example, in Robinson et al. (2013), the authors first summed UPTAGs and DNTAGs for technical replicates within each biological replicate. In contrast, in Romila et al. (2021), the analysis (in this case for the *S. pombe* deletion collection) of the uptags and downtags was considered separately. In a large-scale study from our lab (Lee et al. 2014), we considered the uptag and downtag values independently and selected the “best tag” for each strain for downstream analysis. We found that this approach minimized the noise introduced when a poorly performing tag was averaged with a well-performing tag.

A recent publication from our lab (Barazandeh et al. 2022) illustrates that high-quality data can be derived from considering the uptags and downtags separately or together, but, in light of the fact that the correlation can vary across strains and conditions, we suggest that analyzing each tag separately and using the best tag strategy is advantageous. In Barazandeh et al. (2022), we report that for data generated by our lab, “the HIPLAB dataset” (Lee et al. 2014), the raw data were normalized separately for the strain-specific uptags and downtags. A “best tag” was identified for each strain, defined as the tag with the lowest robust coefficient of variation across all control microarrays. In contrast, in a study of similar size and scope performed by the Novartis Institutes for BioMedical Research (Hoepfner et al. 2014), the uptags and downtags were *averaged* to obtain strain intensity values.

## Results and discussion

### Comparison of FD scores and enriched pathways across sequencing platforms

To assess the reproducibility and consistency of FD scores, we compared results obtained from NovaSeq 6000, NextSeq 550, AVITI, MGI, and Nano-Barseq (Supplementary Fig. 2). FD scores were calculated by normalizing log₂ ratios of tag counts between drug-treated samples and controls, with positive values indicating sensitivity and negative values indicating resistance to the treatment. Different sequencing platforms were tested using different drugs, and selected comparisons are presented here.

**Fig. 4.**
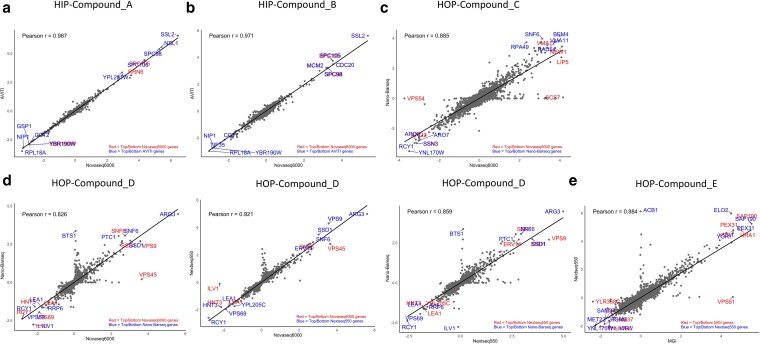
Correlations between FD scores of all genes (up to 6,000/assay) across different sequencing platforms. Each scatter plot compares FD scores obtained from 2 sequencing platforms, with the top and bottom 5 genes labeled. The comparisons include AVITI vs NovaSeq 6000 (2 compounds), Nano-Barseq vs NovaSeq 6000 (2 compounds), NextSeq 550 vs NovaSeq 6000, Nano-Barseq vs NextSeq 550, and MGI vs NextSeq 550. All platforms show at least 82% correlation, with the highest observed between AVITI and NovaSeq 6000 (Pearson *r* = 0.987) and the lowest observed between Nano-Barseq and NovaSeq 6000 for one of the tested compounds (Pearson *r* = 0.826).

Pairwise correlations between platforms (Fig. 4) show that AVITI and NovaSeq 6000 demonstrate the highest agreement (Pearson *r* = 0.987), with high levels of consistency across 2 different drug treatments. NextSeq 550 and NovaSeq 6000 also exhibit high similarity, while Nano-Barseq, although slightly less correlated for one of the examined compounds (Pearson *r* = 0.826), still shows good agreement in top fitness hits. MGI and NextSeq 550 demonstrate an overall correlation above 88%, with some variation among hits but still sharing many top-ranked genes. These results indicate that while some platform-specific differences exist, overall, FD scores are highly reproducible across different sequencing technologies.

To further evaluate the top fitness hits, we examined correlations focusing on the top and bottom (most sensitive and resistant) 50 genes across platforms (Supplementary Fig. 3). Similar trends were observed, with high correlation across all platforms (>83%), and the highest agreement between AVITI and NovaSeq 6000 (Pearson *r* = 0.995).

**Fig. 5.**
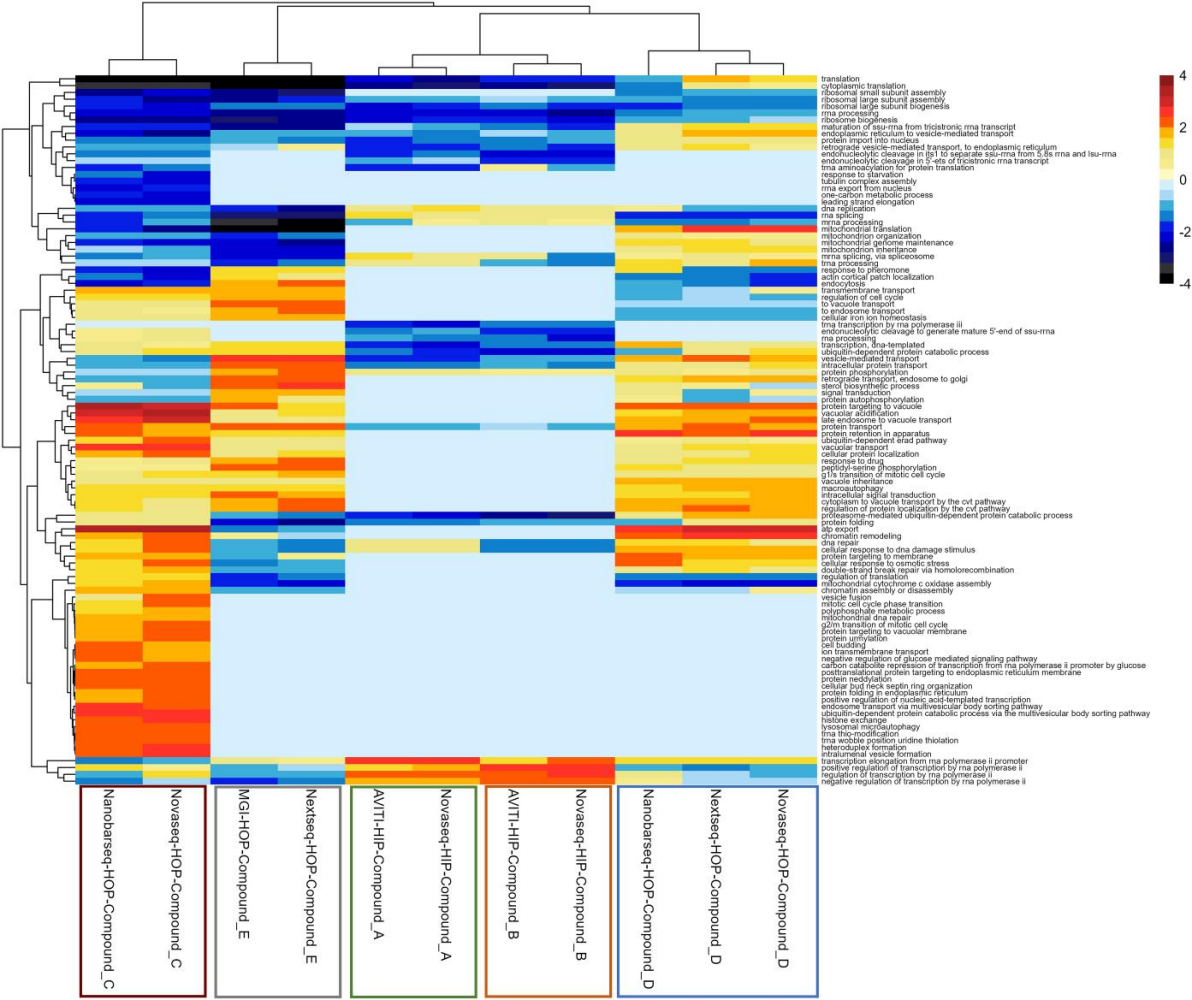
Normalized enrichment scores for GO biological processes across different sequencing platforms, with positive enrichment (red) indicating processes more active in drug-treated conditions compared to control and negative enrichment (blue) indicating processes more suppressed by drug treatment. The platforms compared include NextSeq 550, NovaSeq 6000, AVITI, MGI, and Nano-Barseq, each treated with different drugs. Platforms treated with the same drug cluster together as expected, with most pathways showing consistent enrichment patterns across those platforms.

Finally, Gene Ontology (GO) enrichment analysis (Fig. 5) revealed that data obtained across platforms treated with the same drug clustered together as expected, with most pathways exhibiting consistent enrichment patterns.

Together, these results confirm that Bar-seq data are highly reproducible across sequencing platforms, with only minor variations in hit ranking and enrichment profiles. These small differences may arise from variations in sequencing chemistry, error rates, and amplification biases. Despite this, the strong overall correlations indicate that platform-specific effects do not significantly impact the identification of top-scoring genes and biological conclusions.

### The correlation between uptags and downtags varies across sequencing platforms

**Fig. 6.**
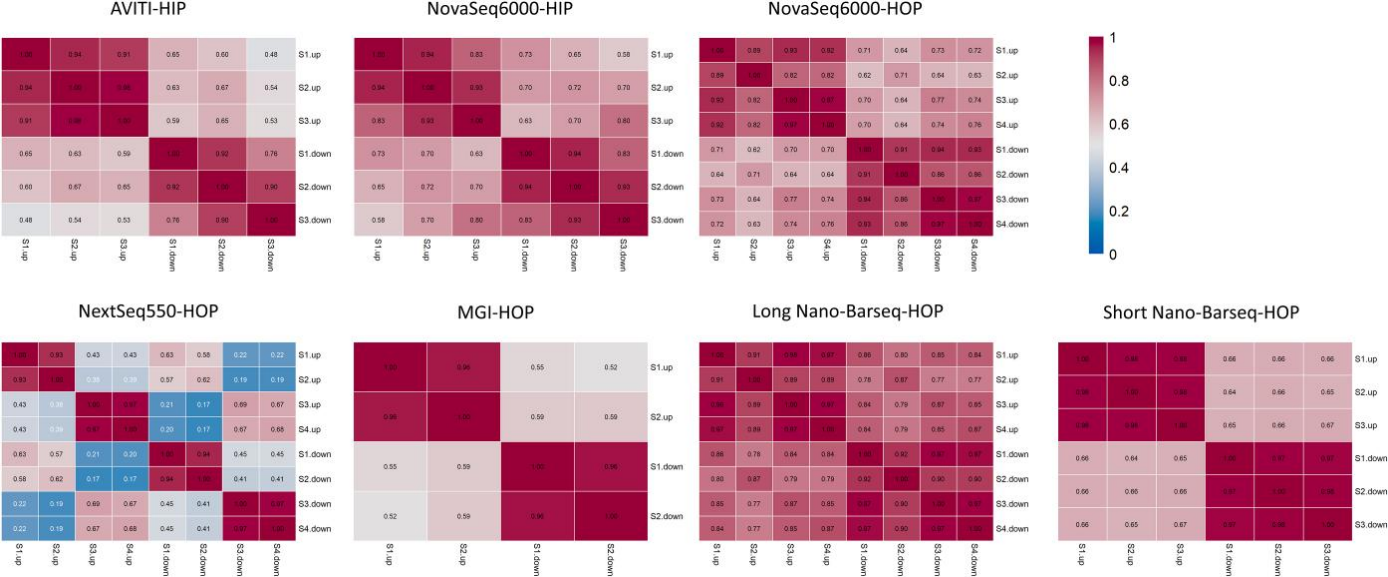
Correlation between uptag and downtag counts across sequencing platforms. Pairwise Pearson correlations are shown for each sample, comparing uptag and downtag counts after averaging across replicates. Each heatmap corresponds to a different sequencing platform. The values represent correlation coefficients between uptags and downtags across thousands of deletion strains in each sample. Two datasets (AVITI and NovaSeq 6000, upper left) are from HIP (heterozygous) screens; all others are from HOP (homozygous) screens.

One of the key features of the Yeast Knockout Collection strains is that they contain 2 unique barcodes, i.e. uptags and downtags, which lie upstream and downstream of the deleted locus. The decision to include 2 tags per strain was made early in the YKO project and was intended to provide redundancy in case one of the tags failed. Accordingly, 97% of all strains contain both an uptag and a downtag (Eason et al. 2004 and http://chemogenomics.pharmacy.ubc.ca/hiplab/GGCN_Lab/SGDP/). In the Bar-seq protocol (with the exception of long Nano-Barseq), the 2 tags are amplified in separate PCR reactions. While, in principle, the signal (or sequence counts) for both tags should be highly correlated, in practice, there is often substantial deviation from perfect correlation. The reasons for this discrepancy are manifold, with differences in PCR amplification being a primary contributor (Pierce et al. 2007). We quantified the correlation between uptags and downtags for each platform tested, analyzing both individual replicates of each sample (Supplementary Fig. 4) and averaged replicates (Fig. 6). A total of 193 of the strains contain only an uptag and lack a corresponding downtag (Supplementary Table 1); these were excluded from the correlation analysis. The correlation values varied; in HIP (heterozygous) experiments, median correlations ranged from 0.65 for the Element platform to 0.73 for the NovaSeq 6000. In HOP (homozygous) experiments, median correlations ranged from 0.57 for MGI, 0.65 for NextSeq 550, 0.66 for short Nano-Barseq, 0.73 for NovaSeq 6000, and 0.87 for long Nano-Barseq. The high correlation for long Nano-Barseq was anticipated because in this protocol, both tags are linked, in cis, to the same molecule. Nevertheless, the correlation was not perfect (maximum *r* = 0.88), possibly due to template switching during the single round of PCR amplification (Stock et al. 2025). This comparison highlights the fact that, when selecting a particular platform for Bar-seq, the uptag–downtag correlation should be factored in.

### The impact of dose and growth inhibition on Bar-seq chemogenomic profiles

To demonstrate the effect of drug dose (and the corresponding growth inhibition of the pools) on the resulting Bar-seq chemogenomic profiles, we focused on a set of samples derived from pools treated with a range of doses of doxycycline, a second-generation, broad-spectrum tetracycline class antibiotic. Specifically, we tested 4 doses of doxycycline using HIPHOP. Additionally, we compared these profiles to those obtained from tetracycline and its structural analogs oxytetracycline, tigecycline, and omadacycline. This work was part of a drug-repurposing effort based on our previous observation that tigecycline has specific effects on mitochondrial translation (Škrtić et al. 2011). All of these antibiotics share a common 4-ring structure, and while their primary mechanism of action is inhibition of bacterial protein synthesis by binding to the 30S ribosomal subunit (Durães and Sousa 2019 ), several studies have explored if they can be useful for new indications, including STI prophylaxis and prevention (Rusu and Buta 2021), inflammation (Di Caprio et al. 2015), and cancer (Barbie and Kennedy 2015).

**Fig. 7.**
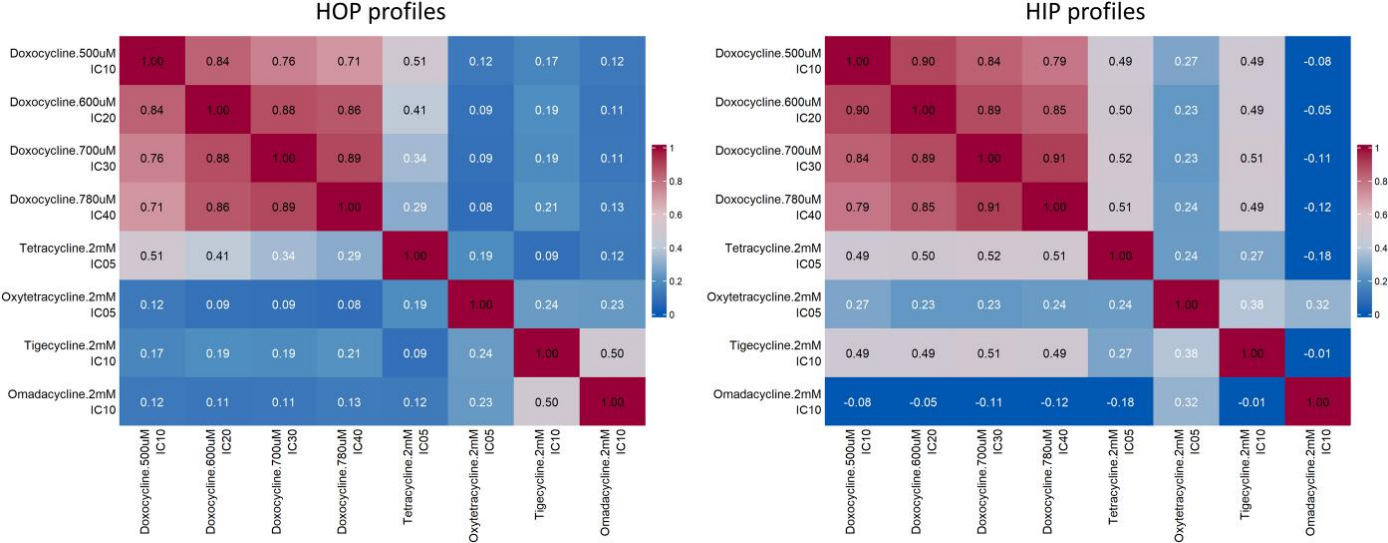
Correlation between Bar-seq chemogenomic profiles across different tetracycline class antibiotics. Pearson correlation heatmaps are shown for pairwise comparisons of FD scores generated using YKO pools treated with increasing doses of doxycycline (500 to 780 µM: IC10 to IC40) and structurally related analogs tetracycline (IC05, 2 mM), oxytetracycline (IC05, 2 mM), tigecycline (IC10, 2 mM), and omadacycline (IC10, 2 mM). Left: Correlations between HOP (homozygous deletion) profiles. Right: Correlations between HIP (heterozygous deletion) profiles. Doxycycline treatments show strong internal consistency across doses (*r* = 0.71 to 0.89 for HOP, *r* = 0.79 to 0.91 for HIP), while structurally similar compounds yield lower correlations even at comparable inhibitory concentrations.

To analyze this data, we generated an aggregate “coinhibition” value for each profile as described in Lee et al. (2014). This analysis shows that for both the nonessential HOP profiles and the essential HIP profiles, the different doses of doxycycline show very high levels of similarity (Fig. 7). Indeed, across a range of 4 doses that inhibit pool growth by 10% to 40%, the correlation between samples ranged from 0.71 to 0.89 for HOP screens and 0.79 to 0.91 for HIP screens. In contrast, the correlation values for structurally similar tetracycline analogs were considerably lower, even for doses that result in similar levels of growth inhibition. For example, in the HOP screen at IC10, the correlation between doxycycline and tigecycline and doxycycline and omadacycline were 0.17 and 0.12, respectively.

### Applications of Bar-seq

Bar-seq has gained prominence in functional genomics due to its high sensitivity, scalability, and cost-effectiveness. Compared to traditional genetic screening methods, Bar-seq enables precise and efficient quantification of strain fitness across large mutant libraries, making it a powerful approach for systematic investigations of gene function and drug interactions. Yet, despite its advantages, it may be daunting for those new to the method to understand and implement the experimental design criteria. Accordingly, we have attempted to synthesize that information in this study.

Our experiments have demonstrated that Bar-seq has broad applications across diverse research fields, such as drug discovery through the identification of drug–target interactions by screening deletion mutants for altered fitness in the presence of small molecules and drugs and functional genomics by systematic mapping of gene essentiality under different environmental conditions. Furthermore, the Bar-seq procedure has been successfully applied to *S. pombe*, demonstrating its utility beyond *S. cerevisiae* (Romila et al. 2021).

### Comparison of sequencing platforms for Bar-seq

Selecting the appropriate sequencing platform is crucial for optimizing Bar-seq experiments. Several sequencing technologies have been employed, each with distinct advantages and limitations (Table 1).

**Table 1.** Comparison of sequencing platforms used for Bar-seq.

Platform	Amplification	Unique advantages	Illumina library compatibility	Max read length	Max throughput/run	Max effective reads/flow cell	>Q30	Cost per million reads (USD)^a^	Best use case
Illumina NextSeq 550	Bridge amplification	Industry standard, mature ecosystem	Yes	2 × 150	120 Gbp	400 M	>85% to 90%	∼12	Large-scale Bar-seq requiring high accuracy
Illumina NovaSeq 6000	Bridge amplification	Ultrahigh-throughput, low cost scalable	Yes	2 × 250	3 Tbp	10 B	>85% to 90%	∼2	Ultrahigh-throughput screening
Oxford Nanopore MinION	None	Ultralong reads, real-time, portable	Yes	Up to 30 kb	48 Gbp	Varies	Not applicable (typically Q20 to Q22 median)	∼50	Portable, real-time Bar-seq analysis
MGISEQ-2000	Rolling circle (DNA nanoballs)	High throughput, low cost, scalable	No	2 × 300	1.44 Tbp	1.8 B	>70% to 85%	∼3 to 4	Illumina alternative
Element AVITI	Rolling circle (PCR-free)	PCR-free, low index hopping, flexible runs	Yes (via Element Adept workflow)	2 × 300	300 Gbp	1 B	>85% to 90%	∼3	Cost-effective alternative to Illumina

^a^The cost/million reads are based on well-established sequencing service providers (e.g. Novogene and Plasmidsaurus) as of July 2025.

Dual indexing of individual samples enables one to run up to 384 unique samples on a single NGS lane; however, to achieve a sufficient number of reads for a pool of 5,000 to 6,000 different barcoded cells, we aim for 5 to 6 million mapped reads per sample. On medium-throughput sequencers, 100 to 200 Bar-seq libraries are typically pooled, whereas higher-throughput platforms (e.g. NovaSeq S4) allow for 384 (or more) samples on a single lane. In practice, we have found that sequencing batches of ∼200 libraries at a time strikes a good balance between throughput and turn-around time.

Nano-Barseq offers the advantage of real-time data generation, eliminating the need for large batch sequencing. Nano-Barseq is compatible with commercial vendors such as Plasmidsaurus (https://plasmidsaurus.com/), which will process pools of amplicons using their rapid turn-around service. Nevertheless, the nanopore platform has a higher error rate compared to short-read platforms, necessitating additional error-correction algorithms. On the other hand, Illumina, AVITI, and MGISEQ platforms provide high accuracy and scalability, making them ideal for large-scale fitness assays.

Ultimately, the choice of sequencing platform depends on the experimental goals, as well as factors such as cost per sample and turn-around time. Studies requiring large sample sizes and precise quantification benefit from the Illumina platform (as well as future ultrahigh-throughput devices such as those from Ultima Genomics), whereas time-sensitive or field-deployable applications may favor Nanopore sequencing.

## Conclusion and future perspectives

Bar-seq has transformed functional genomics by providing a high-throughput, scalable, and cost-effective method for investigating genetic interactions and cellular fitness across diverse research fields. The choice of an appropriate sequencing platform is critical to maximizing the efficiency and accuracy of Bar-seq experiments, with Illumina-based technologies excelling in large-scale studies and Nanopore sequencing offering advantages in real-time analysis. As sequencing technologies continue to evolve, future improvements in read accuracy, cost reduction, and throughput will further expand Bar-seq applications. Bar-seq's simplicity and accessibility make it particularly well-suited for integration with emerging tools in functional genomics.

Another promising direction is the use of Bar-seq to uncover heterogeneity in pooled perturbations at higher resolution. For instance, Bar-seq can be combined with high-throughput cell sorting, such as FACS- or MACS-based selections, followed by barcode counting to identify genetic or phenotypic differences between enriched and depleted populations. This strategy enables the deconvolution of complex traits and responses without the cost or complexity of single-cell sequencing and can be scaled to monitor population-level dynamics across millions of genotypes and thousands of conditions.

In the clinic, the potential of Bar-seq remains untapped. Bar-seq could be applied to patient isolates, e.g. *C. albicans* or diverse bacterial pathogens, to establish drug sensitivity and resistance profiles for patient-specific samples. Finally, applying barcode technology and Bar-seq enumeration on mixed microbial communities promises to accelerate functional genomic studies on the microbiome (Tripathi et al. 2024; Voogdt et al. 2024).

## Supplementary Material

jkaf166_Supplementary_Data

## Data Availability

All sequencing data associated with this study have been deposited in the GenBank Sequence Read Archive (SRA) as BioProject PRJNA1245376 and are available under the accession numbers SAMN47752583 and SAMN47752622. Supplemental material available at G3 online.
