# Supplementary material for: Barcode sequencing: a robust, platform-agnostic method for massively parallel cell-based screens
Source: G3 (Bethesda). 2025 Jul 18;15(9):jkaf166. doi: 10.1093/g3journal/jkaf166 (PMC12405874; doi:10.1093/g3journal/jkaf166)
Supplement: jkaf166_Supplementary_Data [file jkaf166_supplementary_data.zip › Table_S1_G3-2025-406011.pdf]

Table S1. List of 193 YKO deletion strains containing only an uptag and no corresponding downtag.

Unique_uptag	ORF	Gene_Name
YAL001C::chr1_1	YAL001C	TFC3
YAL002W::chr1_1	YAL002W	VPS8
YAL003W::chr1_1	YAL003W	EFB1
YAL004W::chr1_1	YAL004W	YAL004W
YAL005C::chr1_1	YAL005C	SSA1
YAL007C::chr1_1	YAL007C	ERP2
YAL008W::chr1_1	YAL008W	FUN14
YAL009W::chr1_1	YAL009W	SPO7
YAL010C::chr1_1	YAL010C	MDM10
YAL011W::chr1_1	YAL011W	SWC3
YAL013W::chr1_1	YAL013W	DEP1
YAL014C::chr1_1	YAL014C	SYN8
YAL015C::chr1_1	YAL015C	NTG1
YAL017W::chr1_1	YAL017W	PSK1
YAL018C::chr1_1	YAL018C	LDS1
YAL019W::chr1_1	YAL019W	FUN30
YAL020C::chr1_1	YAL020C	ATS1
YAL021C::chr1_1	YAL021C	CCR4
YAL022C::chr1_1	YAL022C	FUN26
YAL023C::chr1_1	YAL023C	PMT2
YAL025C::chr1_1	YAL025C	MAK16
YAL026C::chr1_1	YAL026C	DRS2
YAL027W::chr1_1	YAL027W	SAW1
YAL028W::chr1_1	YAL028W	FRT2
YAL029C::chr1_1	YAL029C	MYO4
YAL030W::chr1_1	YAL030W	SNC1
YAL031C::chr1_1	YAL031C	GIP4
YAL032C::chr1_1	YAL032C	PRP45
YAL033W::chr1_1	YAL033W	POP5
YAL034C::chr1_1	YAL034C	FUN19
YAL034W-A::chr1_1	YAL034W-A	MTW1
YAL035C-A::chr1_1	YAL035C-A	YAL035C-A
YAL035W::chr1_1	YAL035W	FUN12
YAL036C::chr1_1	YAL036C	RBG1
YAL037W::chr1_1	YAL037W	YAL037W
YAL038W::chr1_1	YAL038W	CDC19
YAL039C::chr1_1	YAL039C	CYC3
YAL040C::chr1_1	YAL040C	CLN3
YAL041W::chr1_1	YAL041W	CDC24
YAL042W::chr1_1	YAL042W	ERV46
YAL043C::chr1_1	YAL043C	PTA1
YAL043C-A::chr1_1	YAL043C-A	YAL043C-A
YAL044C::chr1_1	YAL044C	GCV3
YAL045C::chr1_1	YAL045C	YAL045C
YAL046C::chr1_1	YAL046C	BOL3
YAL048C::chr1_1	YAL048C	GEM1

YAL049C::chr1_1	YAL049C	AIM2
YAL051W::chr1_1	YAL051W	OAF1
YAL053W::chr1_1	YAL053W	FLC2
YAL055W::chr1_1	YAL055W	PEX22
YAL056W::chr1_1	YAL056W	GPB2
YAL058W::chr1_1	YAL058W	CNE1
YAL059W::chr1_1	YAL059W	ECM1
YAL060W::chr1_1	YAL060W	BDH1
YAL061W::chr1_1	YAL061W	BDH2
YAL062W::chr1_1	YAL062W	GDH3
YAL065C::chr1_1	YAL065C	YAL065C
YAL066W::chr1_1	YAL066W	YAL066W
YAL067C::chr1_1	YAL067C	SEO1
YAL068C::chr1_1	YAL068C	PAU8
YAR002W::chr1_1	YAR002W	NUP60
YAR003W::chr1_1	YAR003W	SWD1
YAR007C::chr1_1	YAR007C	RFA1
YAR008W::chr1_1	YAR008W	SEN34
YAR014C::chr1_1	YAR014C	BUD14
YAR015W::chr1_1	YAR015W	ADE1
YAR018C::chr1_1	YAR018C	KIN3
YAR019C::chr1_1	YAR019C	CDC15
YAR020C::chr1_1	YAR020C	PAU7
YAR023C::chr1_1	YAR023C	YAR023C
YAR027W::chr1_1	YAR027W	UIP3
YAR028W::chr1_1	YAR028W	YAR028W
YAR029W::chr1_1	YAR029W	YAR029W
YAR030C::chr1_1	YAR030C	YAR030C
YAR031W::chr1_1	YAR031W	PRM9
YAR035W::chr1_1	YAR035W	YAT1
YAR037W::chr1_1	YAR037W	YAR037W
YAR040C::chr1_1	YAR040C	YAR040C
YAR042W::chr1_1	YAR042W	SWH1
YAR043C::chr1_1	YAR043C	YAR043C
YAR044W::chr1_1	YAR044W	YAR044W
YAR047C::chr1_1	YAR047C	YAR047C
YML001W::chr13_2	YML001W	YPT7
YML002W::chr13_2	YML002W	YML002W
YML003W::chr13_2	YML003W	YML003W
YML004C::chr13_2	YML004C	GLO1
YML005W::chr13_2	YML005W	TRM12
YML006C::chr13_2	YML006C	GIS4
YML007W::chr13_2	YML007W	YAP1
YML008C::chr13_2	YML008C	ERG6
YML009C::chr13_2	YML009C	MRPL39
YML010W::chr13_2	YML010W	SPT5
YML010W-A::chr13_2	YML010W-A	YML010W-A
YML011C::chr13_2	YML011C	RAD33
YML012W::chr13_2	YML012W	ERV25

YML013C-A::chr13_2	YML013C-A	YML013C-A
YML013W::chr13_2	YML013W	UBX2
YML014W::chr13_2	YML014W	TRM9
YML015C::chr13_2	YML015C	TAF11
YML016C::chr13_2	YML016C	PPZ1
YML017W::chr13_2	YML017W	PSP2
YML018C::chr13_2	YML018C	YML018C
YML019W::chr13_2	YML019W	OST6
YML020W::chr13_2	YML020W	YML020W
YML023C::chr13_2	YML023C	NSE5
YML024W::chr13_2	YML024W	RPS17A
YML025C::chr13_2	YML025C	YML6
YML026C::chr13_2	YML026C	RPS18B
YML028W::chr13_2	YML028W	TSA1
YML029W::chr13_2	YML029W	USA1
YML030W::chr13_2	YML030W	RCF1
YML032C::chr13_2	YML032C	RAD52
YML033W::chr13_2	YML033W	YML033W
YML034W::chr13_2	YML034W	SRC1
YML035C::chr13_2	YML035C	AMD1
YML035C-A::chr13_2	YML035C-A	YML035C-A
YML037C::chr13_2	YML037C	YML037C
YML043C::chr13_1b	YML043C	RRN11
YML048W::chr13_1b	YML048W	GSF2
YML048W-A::chr13_1b	YML048W-A	YML048W-A
YML049C::chr13_1b	YML049C	RSE1
YML050W::chr13_1b	YML050W	AIM32
YML051W::chr13_1b	YML051W	GAL80
YML052W::chr13_1b	YML052W	SUR7
YML053C::chr13_1b	YML053C	YML053C
YML054C::chr13_1b	YML054C	CYB2
YML055W::chr13_1b	YML055W	SPC2
YML056C::chr13_1b	YML056C	IMD4
YML057W::chr13_1b	YML057W	CMP2
YML058C-A::chr13_1b	YML058C-A	YML058C-A
YML058W::chr13_1b	YML058W	SML1
YML059C::chr13_1b	YML059C	NTE1
YML060W::chr13_1b	YML060W	OGG1
YML061C::chr13_1b	YML061C	PIF1
YML062C::chr13_1b	YML062C	MFT1
YML063W::chr13_1b	YML063W	RPS1B
YML064C::chr13_1b	YML064C	TEM1
YML065W::chr13_1b	YML065W	ORC1
YML078W::chr13_1b	YML078W	CPR3
YML079W::chr13_1b	YML079W	YML079W
YML080W::chr13_1b	YML080W	DUS1
YML081W::chr13_1b	YML081W	TDA9
YML082W::chr13_1b	YML082W	YML082W
YML083C::chr13_1b	YML083C	YML083C

YML084W::chr13_1b	YML084W	YML084W
YML086C::chr13_1b	YML086C	ALO1
YML087C::chr13_1b	YML087C	AIM33
YML088W::chr13_1b	YML088W	UFO1
YML089C::chr13_1b	YML089C	YML089C
YMR001C::chr13_2	YMR001C	CDC5
YMR002W::chr13_2	YMR002W	MIX17
YMR003W::chr13_2	YMR003W	AIM34
YMR005W::chr13_2	YMR005W	TAF4
YMR006C::chr13_2	YMR006C	PLB2
YMR007W::chr13_2	YMR007W	YMR007W
YMR008C::chr13_2	YMR008C	PLB1
YMR009W::chr13_2	YMR009W	ADI1
YMR010W::chr13_2	YMR010W	ANY1
YMR011W::chr13_2	YMR011W	HXT2
YMR012W::chr13_2	YMR012W	CLU1
YMR013C::chr13_2	YMR013C	SEC59
YMR014W::chr13_2	YMR014W	BUD22
YMR015C::chr13_2	YMR015C	ERG5
YMR016C::chr13_2	YMR016C	SOK2
YMR017W::chr13_2	YMR017W	SPO20
YMR018W::chr13_2	YMR018W	PEX9
YMR019W::chr13_2	YMR019W	STB4
YMR020W::chr13_2	YMR020W	FMS1
YMR021C::chr13_2	YMR021C	MAC1
YMR022W::chr13_2	YMR022W	UBC7
YMR023C::chr13_2	YMR023C	MSS1
YMR024W::chr13_2	YMR024W	MRPL3
YMR025W::chr13_2	YMR025W	CSI1
YMR026C::chr13_2	YMR026C	PEX12
YMR027W::chr13_2	YMR027W	YMR027W
YMR028W::chr13_2	YMR028W	TAP42
YMR029C::chr13_2	YMR029C	FAR8
YMR030W::chr13_2	YMR030W	RSF1
YMR031C::chr13_2	YMR031C	EIS1
YMR031W-A::chr13_2	YMR031W-A	YMR031W-A
YMR032W::chr13_2	YMR032W	HOF1
YMR033W::chr13_2	YMR033W	ARP9
YMR034C::chr13_2	YMR034C	RCH1
YMR035W::chr13_2	YMR035W	IMP2
YMR036C::chr13_2	YMR036C	MIH1
YMR038C::chr13_2	YMR038C	CCS1
YMR039C::chr13_2	YMR039C	SUB1
YMR040W::chr13_2	YMR040W	YET2
YMR041C::chr13_2	YMR041C	ARA2
YMR042W::chr13_2	YMR042W	ARG80
YMR043W::chr13_2	YMR043W	MCM1
YMR044W::chr13_2	YMR044W	IOC4
YMR047C::chr13_2	YMR047C	NUP116
