# Supplementary figures and images for: Barcode sequencing: a robust, platform-agnostic method for massively parallel cell-based screens
Source: G3 (Bethesda). 2025 Jul 18;15(9):jkaf166. doi: 10.1093/g3journal/jkaf166 (PMC12405874; doi:10.1093/g3journal/jkaf166)

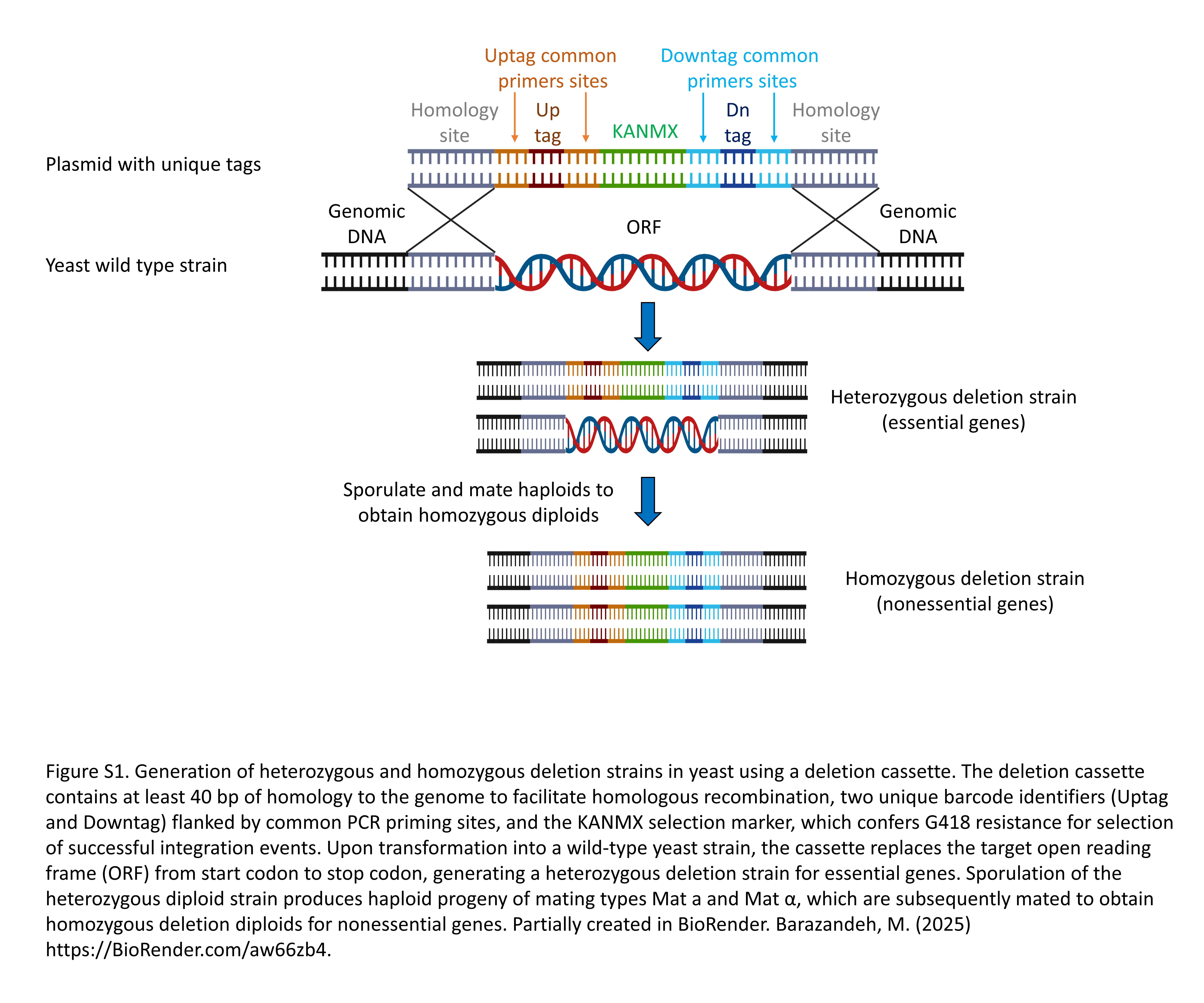

Supplement: jkaf166_Supplementary_Data [file jkaf166_supplementary_data.zip › Figure_S1_G3-2025-406011.png]

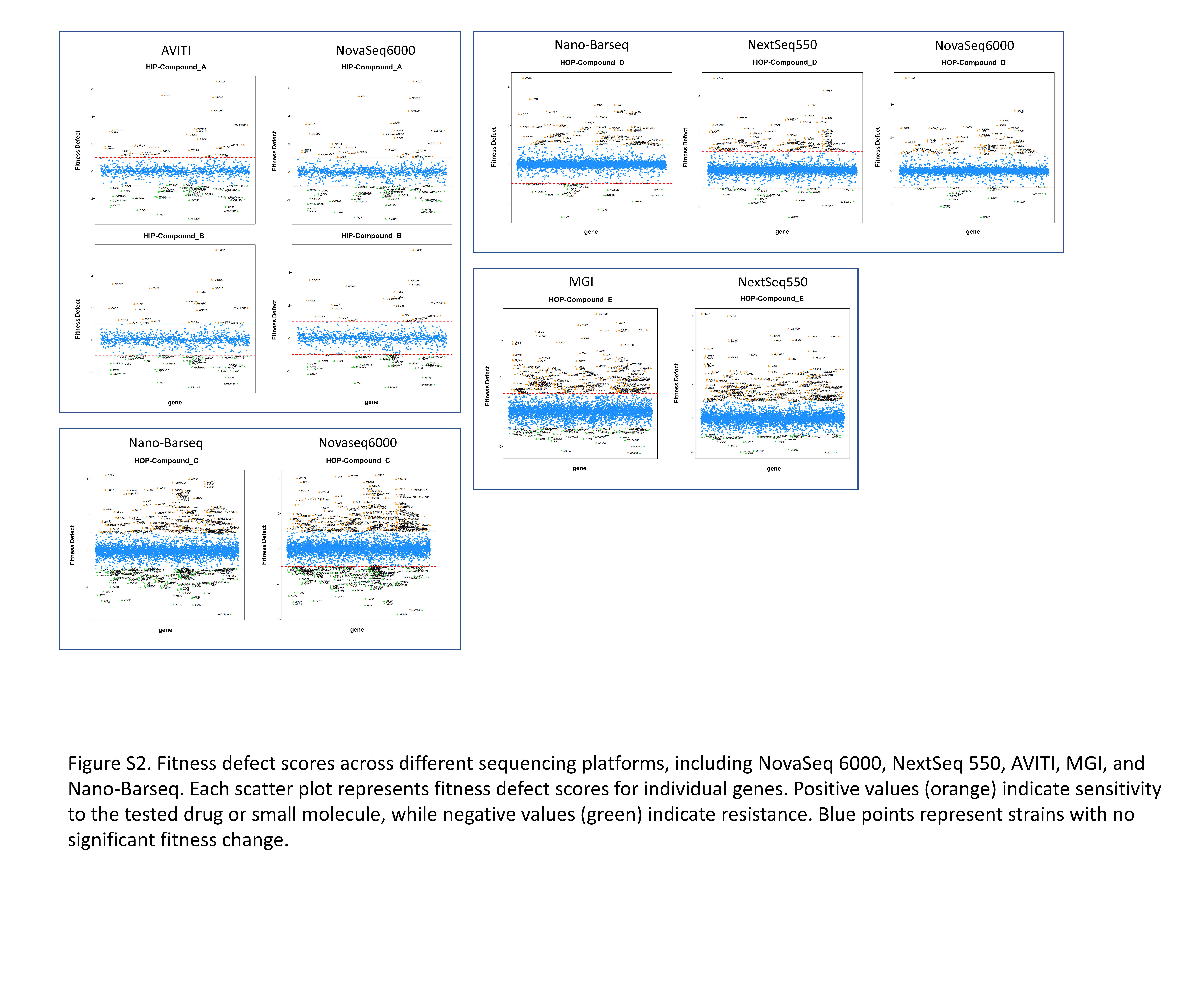

Supplement: jkaf166_Supplementary_Data [file jkaf166_supplementary_data.zip › Figure_S2_G3-2025-406011.png]

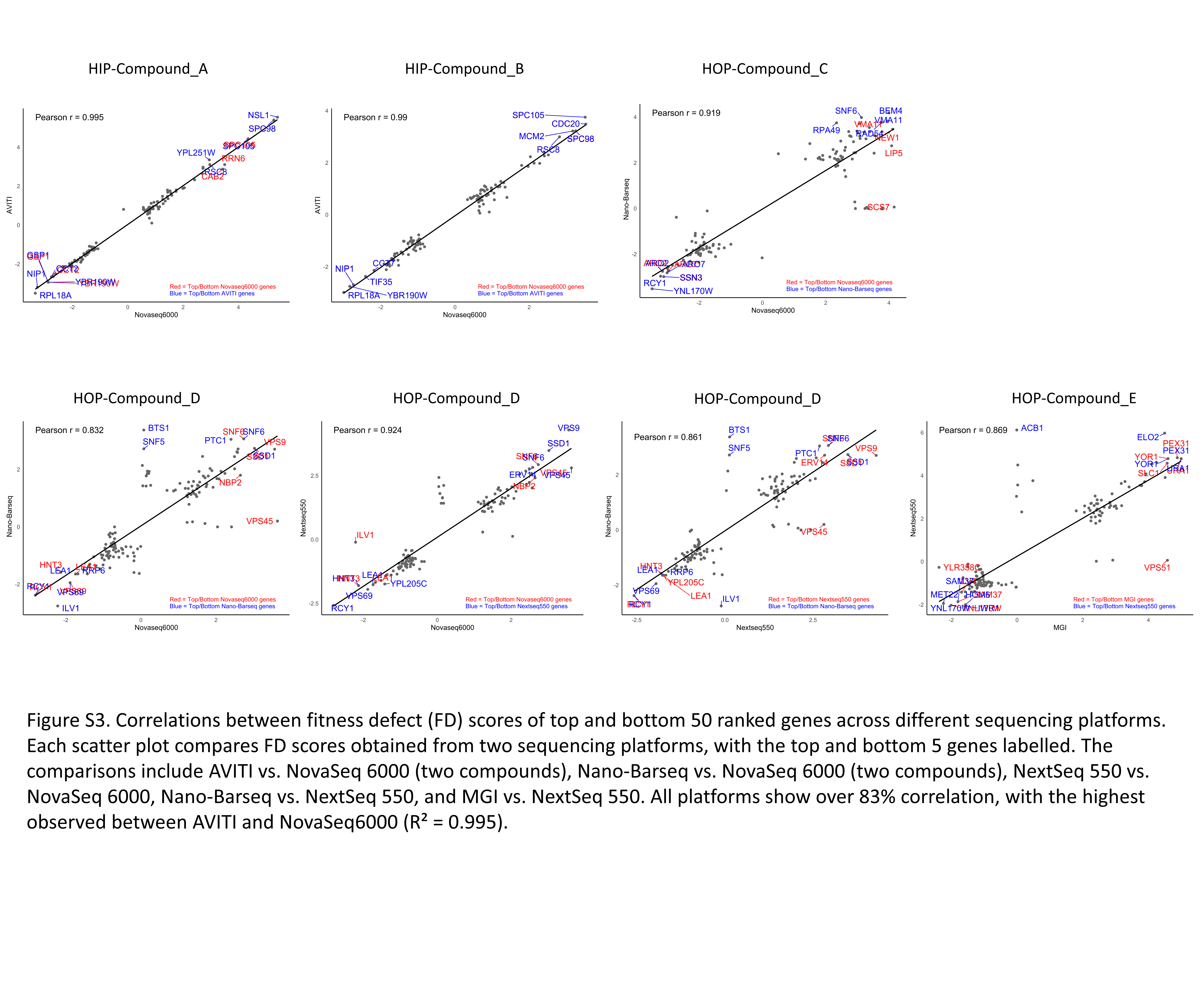

Supplement: jkaf166_Supplementary_Data [file jkaf166_supplementary_data.zip › Figure_S3_G3-2025-406011.png]

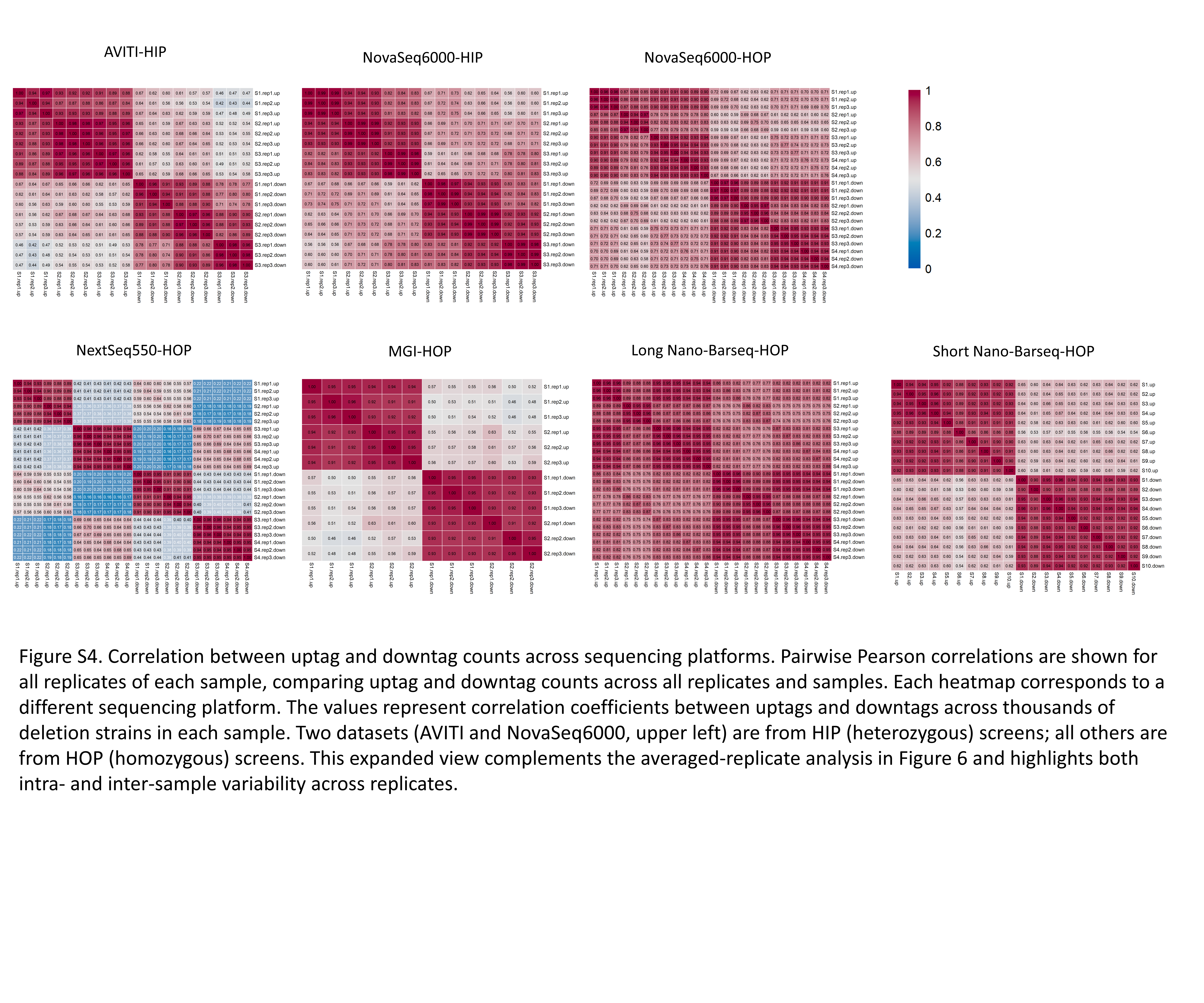

Supplement: jkaf166_Supplementary_Data [file jkaf166_supplementary_data.zip › Figure_S4_G3-2025-406011.png]
